# Wind-Wave Synergistic Triboelectric Nanogenerator: Performance Evaluation Test and Potential Applications in Offshore Areas

**DOI:** 10.3390/mi15030314

**Published:** 2024-02-24

**Authors:** Zhen Pan, Weijian Wu, Jiangtao Zhou, Yili Hu, Jianping Li, Yingting Wang, Jijie Ma, Jianming Wen

**Affiliations:** 1 The Institute of Precision Machinery and Smart Structure, College of Engineering, Zhejiang Normal University, Yinbin Street 688, Jinhua 321004, China; pz72988@zjnu.edu.cn (Z.P.); wuweijian@zjnu.edu.cn (W.W.); zhoujiangtao@zjnu.edu.cn (J.Z.); huyili@zjnu.edu.cn (Y.H.); lijp@zjnu.cn (J.L.); wjming@zjnu.cn (J.W.); 2 Key Laboratory of Intelligent Operation and Maintenance Technology & Equipment for Urban Rail Transit of Zhejiang Province, Zhejiang Normal University, Yinbin Street 688, Jinhua 321004, China

**Keywords:** triboelectric nanogenerator, ocean energy collection, wind-wave synergistic, self-power

## Abstract

Triboelectric nanogenerators (TENGs) can effectively collect low-frequency, disordered mechanical energy and are therefore widely studied in the field of ocean energy collection. Most of the rotary TENGs studied so far tend to have insufficient rotation, resulting in slow charge transfer rates in low-frequency ocean environments. For this reason, in this paper, we propose a wind-wave synergistic triboelectric nanogenerator (WWS-TENG). It is different from the traditional rotary TENGs based on free-standing mode in that its power generation unit has two types of rotors, and the two rotors rotate in opposite directions under the action of wind energy and wave energy, respectively. This type of exercise can more effectively collect energy. The WWS-TENG has demonstrated excellent performance in sea wind and wave energy harvesting. In the simulated ocean environment, the peak power can reach 13.5 mW under simulated wind-wave superposition excitation; the output of the WWS-TENG increased by 49% compared to single-wave power generation. The WWS-TENG proposal provides a novel means of developing marine renewable energy, and it also demonstrates broad application potential in the field of the self-powered marine Internet of Things (IoT).

## 1. Introduction

In the modern world, the problems of global climate change, energy shortage, and environmental pollution have increasingly attracted great attention from governments, international organizations, and the general public [1]. In order to effectively solve the increasingly serious energy and environmental problems, it has become a global consensus to vigorously develop renewable and clean energy [2]. The marine environment has huge energy reserves, such as sea wind energy, wave energy, tidal energy, and other clean energy [3]. Hence, the creation of a technology with which to efficiently extract clean energy from the ocean is crucial for modern society. The triboelectric nanogenerator (TENG) is a device that utilizes the coupling effect of triboelectrification and electrostatic induction to convert mechanical energy into electrical energy [4]. TENGs are advantageous due to their light weight, their simple structure, the availability of various material options, and their low cost when compared to traditional electromagnetic generators, and they can more effectively collect weak and low-frequency mechanical energy [5], such as human kinetic energy [6,7,8], wind energy [9,10,11,12,13], wave energy [14,15,16,17], and water energy [18,19]. Consequently, TENGs will become a promising and efficient harvesting technology for converting wave and wind energy into electrical power in the marine environment.

In recent years, multiple types of TENG with diverse structures and operating modes have become available for capturing wave energy. Common structures generally include rolling ball or column structures [20,21,22,23,24,25] based on free-standing mode, stacked layer structures [26,27,28,29] based on contact-separation mode, cylindrical pendulum structures [30,31,32], and rotating disk or cylindrical structures [33,34,35] based on free-standing mode. Rui et al. presented a high-performance cylindrical pendulum-shaped triboelectric nanogenerator (CP-TENG); the arched FEP film’s design increases the contact area, leading to the excellent performance of the CP-TENG under resonance conditions [36]. However, if the excitation frequency deviates from the resonance frequency, the stability of the output energy and the energy conversion efficiency of the CP-TENG will be drastically reduced. Han et al. proposed a cylindrical wave-driven linkage mechanism triboelectric nanogenerator (WLM-TENG) with unidirectional rotation [37]; the WLM-TENG, driven by enormous buoyancy force, showed excellent output performance, and its peak power could reach 50 mW. Jiang et al. presented a TENG based on a screw rod and ratchet design (SR-TENG) [38]. It enables the rotor to rotate in one direction for positive excitation energy collection and accumulation, and the frequency of electrical output was increased. These unidirectional rotating fixed-type TENGs can address the traditional CP-TENG application defects, but these TENGs are affected by wave height and frequency, their rotor rotation is not sufficient, and the charge transfer rate will be slow at low frequencies. Hence, achieving higher rotational frequencies to accelerate the charge transfer rate is the key to improving the power generation performance.

According to the wind direction (from the ocean to the land) and wind speed (generally 5–7 m/s) characteristics of the sea wind, this paper proposes a wind-wave synergistic triboelectric nanogenerator (WWS-TENG). The WWS-TENG is different from the traditional rotary TENG based on free-standing mode; the WWS-TENG unit has two rotors, which are driven by waves and sea wind, respectively, to achieve unidirectional rotation, and the two rotors rotate in opposite directions. At present, most TENGs applied in the marine environment can only collect single-wave energy or wind energy, and the collection method is relatively simple; this structural design of the WWS-TENG not only realizes the synergistic collection of wave and wind energy, expands the range of energy collection, and improves scalability; is also effectively combines the two types of energy so that the two rotors of the generator rotate in a manner more fully relative to each other, which greatly accelerates the rate of charge transfer and improves the output performance of the generator. In the simulated ocean environment, the WWS-TENG can reach peak power of 13.5 mW under simulated wind-wave superposition excitation. The output of the WWS-TENG increased by 49% compared to single-wave power generation. This work effectively addresses the problem of insufficient rotation of the traditional rotary TENG based on free-standing mode for harvesting ocean energy, provides a theoretical basis for high-power TENG for harvesting wave and sea wind energy, and also provides critical power support for the low-power sensors in self-powered marine Internet of Things (IoT) applications.

## 2. Materials and Methods

### 2.1. Fabrication of the WWS-TENG

The WWS-TENG is fixed on an acrylic base plate. The dimensions of the acrylic base plate are 560 mm × 300 mm × 20 mm (length × width × height). The blades of the wind energy input module are made by a 3D printer, the printed material being polylactic acid (PLA). The swinging lever is made of wood and is fixed on the output shaft of the driving gears. The spherical buoy is fixed on the swinging lever. Two driving gears and two one-way gears are made by a 3D printer. The number of teeth of the two driving gears is 100, and both gears have the module of 1. The number of teeth of the two one-way gears is 20, and the driving gears and the one-way gears have the same module of 1. A one-way bearing is mounted in the center of each one-way gear to ensure the one-way transmission of torque. The two power generation units are placed in opposite directions. The diameter of the PCB with copper electrodes is 150 mm, and the thickness of the PCB is 1.6 mm. The diameter of the acrylic disc with fluorinated ethylene propylene (FEP) is also 150 mm, and the thickness of the disc is 2 mm. The thickness of the FEP film is 0.05 mm.

### 2.2. Experimental Procedure and Measuring Equipment

For performance testing of the WWS-TENG, a rotary motor and a blower were used to create a simulated test system. The power generation unit’s output performance was tested using a programmable electrometer (Keithley Model 6514, Cleveland, OH, USA), and the data were collected using a data acquisition system (PCI-6259, National Instruments, Louisville, CO, USA). The collected signals are recorded by the LabVIEW (2019 version) program.

## 3. Results and Discussion

### 3.1. The Structure of WWS-TENG

**Figure 1:**
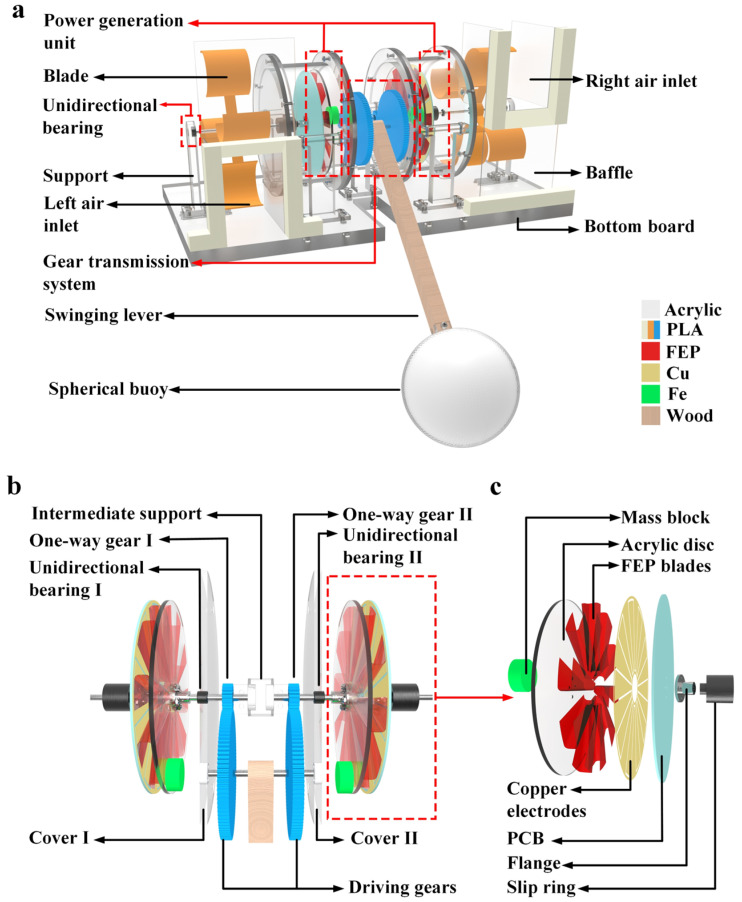
Diagram depicting the structure of WWS-TENG: (**a**) overall structure schematic diagram of WWS-TENG; (**b**) detailed drawing of gear transmission system; (**c**) detailed drawing of power generation unit.

Figure 1a illustrates the structure of WWS-TENG, which includes two wind energy input modules, a gear transmission system, two power generation units, a swinging lever, and a spherical buoy. The two wind energy input modules are located on one side of the corresponding power generation unit, which include the blades, the air inlet, and the support with the unidirectional bearing such that the support base ensures that the output shaft always rotates in one direction. Figure 1b provides a detailed view of the gear transmission system. The transmission ratio of the gear transmission system is 5. The two driving gears and the wooden swinging lever are fixed on the input shaft. The two driven gears are one-way gear I and one-way gear II, respectively, which are located on the two sides of the intermediate support and are fixed to two corresponding center shafts, and the installation direction of the two one-way gears is opposite, so the rotation direction of the two center shafts also is opposite. Two bearings, unidirectional bearing I and unidirectional bearing II, are mounted on cover I and cover II, respectively, and are also fixed to two corresponding center shafts. The installation directions of the one-way gear I and unidirectional bearing I are the same. Similarly, the installation directions of the one-way gear II and unidirectional bearing II are also identical. This means that the two corresponding center shafts can only rotate in one direction and lock in the opposite direction. The construction of the power generation unit is presented in Figure 1c. The power generation unit consists primarily of a mass block, an acrylic disc with fluorinated ethylene propylene (FEP) blades, a printed circuit board (PCB) that is attached with copper electrodes, and an electrically conductive slip ring. The number of copper electrode pairs on the PCB is 10 pairs, and the number of fluorinated ethylene propylene (FEP) blades on the acrylic disc is 10. The mass block is attached to the acrylic disc with FEP blades, which mainly plays the role of gravity energy storage. The conductive slip ring is located on one side of the PCB, mainly to solve the PCB wiring problem. The sea wind passes through the air inlets on both sides, blowing the blades to rotate, thus driving the PCB with copper electrodes to rotate. The undulating motion of the waves will push the swinging lever connected with the spherical buoy to swing and drive the acrylic disc with FEP blades to rotate unidirectionally under the influence of the gear transmission system. The rotation direction of the acrylic disc and corresponding PCB is opposite in same power generation unit; this type of movement also allows the WWS-TENG to realize the simultaneous harvesting of sea wind and wave energy, and due to the use of the 0.05 mm-thick FEP blades and the sealing of the power generation unit with an acrylic shell, the power generation unit has less wear and does not come into contact with seawater; thus, the WWS-TENG will have good durability and reliability at various water depths and salinities.

### 3.2. The Working Principle of WWS-TENG

**Figure 2:**
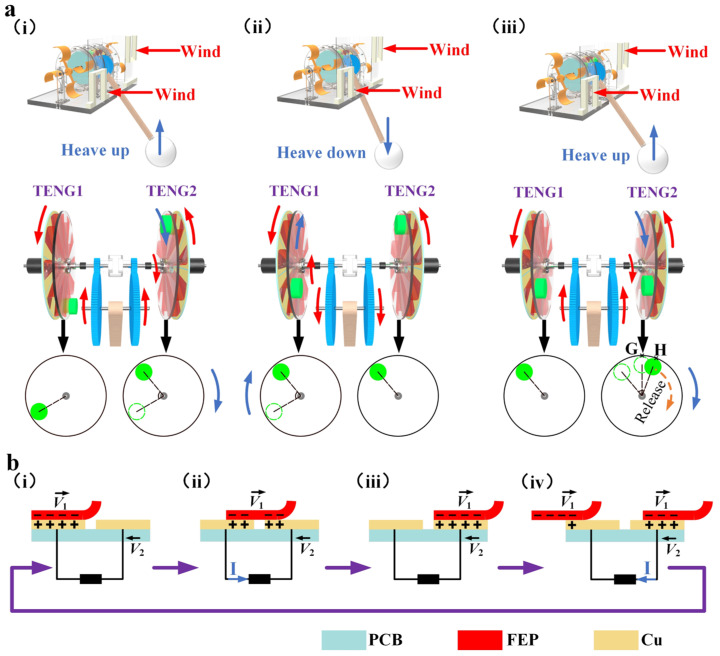
Working principle of WWS-TENG: (**a**) the energy storage–release process of the mass block of the WWS-TENG; (**b**) the power generation principle of WWS-TENG.

Figure 2a roughly depicts the energy storage–release process of the mass block during the collection of wind and wave energy by WWS-TENG. As shown in Figure 2a(i), when the wave rises, the spherical buoy will be heaved up, which makes the driving gears of gear transmission system rotate upward. At this time, the one-way gear II and the center shaft on the right side of the middle support rotate together, thus driving the right side of the acrylic disc with FEP blades and the mass block to rotate. The mass block has not reached the highest point at that moment; the acrylic disc and the corresponding PCB rotate in the opposite direction. However, at the same moment, the one-way gear I on the left side will not drive the center shaft on the left side to rotate, and only the sea wind will drive the PCB to rotate. At this stage, the mass block of the right TENG2 is at the energy storage stage, while the mass block of the left TENG1 is at the stationary state. Force analysis and the moment balance equation at the stage are depicted in Figure S1 and Note S1. Similarly, when the wave descends (Figure 2a(ii)), the swinging lever will swing downward under the influence of gravity, and the spherical buoy will be heaved down, causing the driving gears to rotate downward, which, in turn, drives only the acrylic disc on the left side to rotate. At this stage, the mass block of the left TENG1 is at the energy storage stage, while the mass block of the right TENG2 is in the stationary state. As shown in Figure 2a(iii), the right acrylic disc will continue to rotate when the wave rises again. When the mass block of the right acrylic disc rotates to the highest point G, the mass block’s gravitational potential energy is the largest at this point, but the gravity of the mass block at this point produces a torque of 0 towards the center of rotation. As the acrylic disc continues to rotate, the torque generated by the gravity of the mass block becomes progressively larger. Assuming that the mass block rotates to point H, the torque generated by the gravity of the mass block is just greater than the friction torque acting on the FEP blades; at this point, even without additional external excitation, the acrylic disc can rotate even under the action of the gravitational potential energy of the mass block. We call point H the energy release point. Figure S2 and Note S2, respectively, show the force analysis and moment balance equation at the stage. When the wave descends and allows the left mass block to reach the energy release point, the left acrylic disc will also rotate under the influence of gravity potential energy. In subsequent experiments, we also found that when the oscillation (45°) frequency is greater than 1.5 Hz, the left and right acrylic disc changes from this intermittent rotation to continuous rotation. 

Figure 2b describes the power generation principle of WWS-TENG. As shown in Figure 2b(i), it is assumed that the copper electrode and the FEP blade are completely overlapped in the initial state, at which time the contact surface of the copper electrode carries positive charges, while the contact surface of the FEP blade carries negative charges. As the FEP blade rotates to the right and the PCB with copper electrodes rotates in the opposite direction to the rotation of the FEP blade, the negative charges on the contact surface of the FEP blade will cause the positive charges on the left copper electrode to transfer to the right copper electrode, this results in an electric current being generated (Figure 2b(ii)). When the right copper electrode is fully covered by the FEP blade, all the positive charges on the left copper electrode are completely transferred to the right copper electrode (Figure 2b(iii)). Then, when the other FEP blade rotates to the left copper electrode, the positive charge of the right copper electrode will be transferred to the left copper electrode, resulting in the generation of an electric current once more (Figure 2b(iv)).

### 3.3. Output Performance of WWS-TENG

#### 3.3.1. The Output Performance under Simulated Sea Wind Excitation

**Figure 3:**
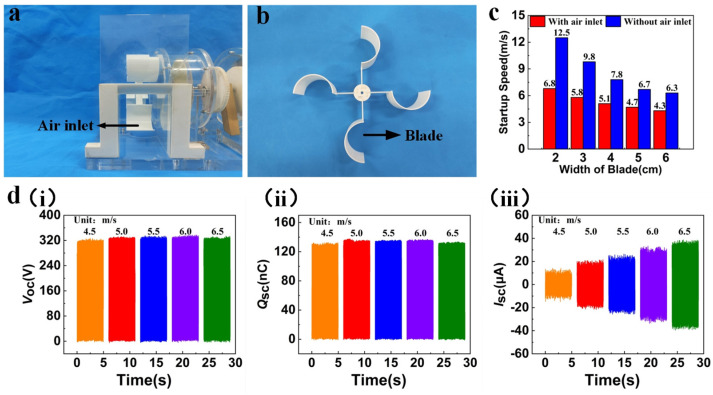
The output performance under simulated sea wind excitation: (**a**) photographs of the air inlet; (**b**) photographs of the blades; (**c**) relationship between blade width and startup wind speed; (**d**) output performance of a single power generation unit at different wind speeds.

In order to adapt the generator to the wind speed range of the sea wind, we optimized the wind input port and blade width. Given the differing specific heat capacities of the ocean and land, a sea wind consistently flows from the sea to the land. To capitalize on this environmental characteristic, we positioned a specifically designed air inlet at the blade’s front, as illustrated in Figure 3a. Blades with different widths (2 cm, 3 cm, 4 cm, 5 cm, and 6 cm) were fabricated by the 3D printer, as depicted in Figure 3b. Startup wind speeds were compared with and without air inlets at different blades, respectively. The data demonstrating the relationship between blade width and wind speed at startup is depicted in Figure 3c. The study reveals that as the blade width increases, the startup wind speed, both with and without an air inlet, undergoes a progressive decline. Specifically, with the increase in blade width from 2 cm to 6 cm, the startup wind speed decreases from 6.8 m/s to 4.3 m/s for the model with the air inlet, and from 12.5 m/s to 6.3 m/s for the model without the air inlet. Most notably, in the case of the 6 cm blade width, the startup wind speed with the air inlet decreases by about 31.7% compared to that without the air inlet. This is mainly due to the fact that one end of the wind turbine is blocked by the corresponding baffle, so the wind can only be blown in through the inlet, and the larger blade width also increases the windward area, which, in turn, increases the torque. With this rational design, the wind energy in the environment can be captured effectively. The output performance of the WWS-TENG relies on its power generation unit that is the essential element of the WWS-TENG. To analyze the effectiveness of the WWS-TENG for collecting sea wind energy, we studied the wind speed range of the actual sea wind (the annual average wind speed in the coastal area of South Jiangsu, China, is typically within 6.5 m/s), and adopted a blower to simulate the sea wind in the real environment. The wind speed range of the blower is set at 4.5 m/s to 6.5 m/s to evaluate the performance of a single generator unit at various wind speeds.

Figure 3d illustrates the output performance of a single power generation unit at different wind speeds. Significantly, the open-circuit voltage (Figure 3d(i)) and transferred charge (Figure 3d(ii)) exhibit less fluctuation and maintain relative stability as wind speed escalates from 4.5 m/s to 6.5 m/s. The open-circuit voltage stabilized at about 323 V, while the transferred charge retained approximately 127 nC. In contrast, the short-circuit current (Figure 3d(iii)) increased from 14.2 μA to 38.7 μA, changing in an almost linear fashion. The fundamental factor contributing to this phenomenon is that higher wind speed enhances rotational speed, subsequently influencing power generation performance. Open-circuit voltage and transferred charge of the WWS-TENG demonstrate a less significant relationship with the relative rotational speed and are related to the contact area. Conversely, increasing the rotational speed will result in a higher charge transfer rate and, consequently, raise the short-circuit current. In summary, the WWS-TENG can effectively capture sea wind energy, and output performance increases with wind speed.

#### 3.3.2. The Output Performance under Simulated Wave Excitation

**Figure 4:**
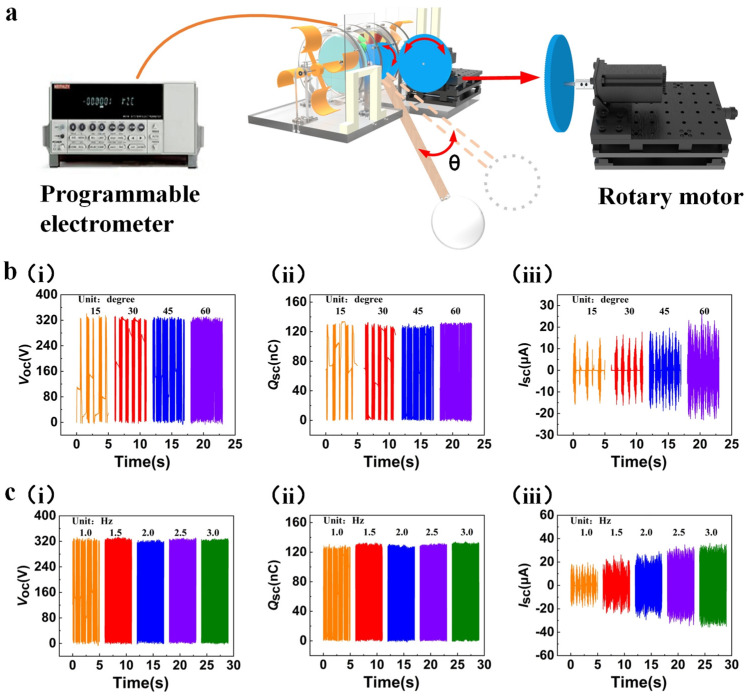
The output performance under simulated wave excitation: (**a**) simulating the operating status of the WWS-TENG via driving rotary motor; (**b**) output performance of single power generation unit at different swing angles; (**c**) output performance of a single power generation unit at different frequencies.

In a real coastal environment, the buoy of the WWS-TENG would be placed on the surface of the sea. The undulation of waves makes the buoy stationed on the swinging lever rise or fall, and the swinging lever generates the corresponding swing simultaneously. The oscillating swinging lever will drive the acrylic disc with FEP blades and the mass block through the gear transmission system to rotate in one direction, which will cause the WWS-TENG to generate electricity. In order to simulate the working condition of the WWS-TENG when it is impacted by water waves in the real environment, a controllable rotary motor that is set to swing mode is used to drive the gear transmission system to rotate to assess the output performance of the WWS-TENG under wave excitation (Figure 4a). The output shaft of the rotary motor has a driving gear that has the teeth number of 150, the driving gear and the gear of the gear transmission system have the same module of 1, and the transmission ratio of these two gears is 1.5. Therefore, the swing angle of swinging lever is 1.5 times the swing angle of the rotary motor. The swing angle of swinging lever was set to range from 15° to 60° and the frequency was set to 1 Hz to explore the output performance of a single power generation unit under different swing angles, as depicted in Figure 4b. It was noted that the open-circuit voltage (Figure 4b(i)) remained approximately constant at around 325 V, and the transferred charge (Figure 4b(ii)) also showed negligible variation, hovering at approximately 125 nC. In contrast, the short-circuit current (Figure 4b(iii)) exhibited a noticeable increase from 16.5 μA to 26.4 μA. The reason for this phenomenon is that at a fixed frequency, an increase in the swing angle results in a relevant rise in the angular velocity, consequently leading to an augmented short-circuit current. Additionally, the elevation of the swing angle results in a reduction of the energy storage time for the mass block, while also extending the continuous rotation time after reaching the energy release point.

Furthermore, the rotary motor’s swing angle was set at 30° (45° for the swinging lever), and the corresponding frequencies were 1 Hz, 1.5 Hz, 2.0 Hz, 2.5 Hz, and 3.0 Hz to study the performance output of a single power generation unit under different frequencies. As illustrated in Figure 4c, as the frequency increases from 1.0 Hz to 3.0 Hz, the open-circuit voltage (Figure 4c(i)) and transferred charge (Figure 4c(ii)) are almost unchanged, and there was an increase in short-circuit current (Figure 4c(iii)) from 19.6 μA to 36.1 μA, with an approximately linear increase in the rate of output charge. The reason for this phenomenon is similar to what we analyzed before; the angular velocity of the acrylic disc increases with frequency when the swing angle is fixed. At the same time, when the frequency is lower (≤1 Hz), the energy stored in the acrylic disc with the mass block is lower and would be consumed quickly by friction. The acrylic disc intermittently rotates, but when the frequency is higher (≥1.5 Hz), the acrylic disc can obtain more energy and continuously rotate. Figures S3–S5 show comparisons of output performance curves under intermittent rotation and continuous rotation.

#### 3.3.3. The Output Performance under Simulated Wind-Wave Superposition Excitation

**Figure 5:**
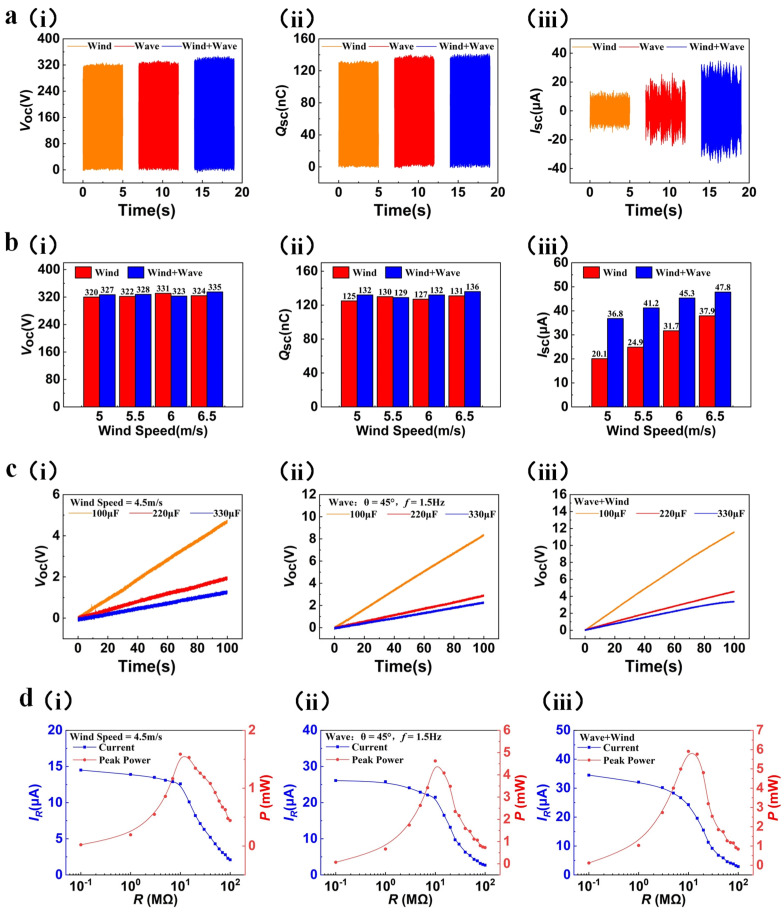
The output performance under simulated wind-wave superposition excitation: (**a**) the output performance of a single power generation unit under simulated wind-wave superposition excitation; (**b**) comparison of output performance under simulated wind-wave superposition excitation with different wind speeds; (**c**) voltage of different load capacitors under different external excitations; (**d**) output power and output current of a single power generation unit under different external excitations.

To study the output performance of a single power generation unit under the wind-wave superposition excitation, we used a blower to simulate the sea wind excitation and used a rotary motor to simulate the wave excitation. The wind speed was adjusted to 4.5 m/s and the swing angle of the swinging lever was adjusted to 45° (1.5 Hz) for this purpose. An acrylic disc with FEP blades and a mass block and a PCB with copper (Cu) electrodes are rotated in opposite directions under two kinds of external excitation generated by a blower and a rotating motor, respectively. Based on the results displayed in Figure 5a, the open-circuit voltage (Figure 5a(i)) and transferred charge (Figure 5a(ii)) of a single power generation unit under the simulated wind-wave superposition excitation do not significantly increase compared to those under simulated sea wind excitation or wave excitation. Notably, the short-circuit current (Figure 5a(iii)) can achieve a value of 34.7 μA, which represents an increase of about 144% and 32% compared to that obtained under simulated sea wind excitation or wave excitation, respectively.

The reasons for these results are similar to those of the results previously discussed. The reverse rotation of the PCB and acrylic disc increases the relative speed of rotation compared to the rotation of a single PCB or acrylic disc, leading to a significant increase in the rate of charge output and short-circuit current. We still set the swing angle of the swinging lever to 45° (1.5 Hz); then, we test the output performance of the power generation unit under the simulated wind-wave superposition excitation with different wind speeds to verify the reliability of our analysis. As shown in Figure 5b, as the wind speed rises from 5.0 m/s to 6.5 m/s, the open-circuit voltage (Figure 5b(i)) and transferred charge (Figure 5b(ii)) under the simulated wind-wave superposition excitation are still relatively stable, and the short-circuit current (Figure 5b(iii)) rises from 36.8 μA to 47.8 μA, which is significantly higher compared to that under simulated sea wind excitation or wave excitation. Such experimental phenomena also further verify the reliability of our analysis. In order to further investigate the output performance of the WWS-TENG, we test a single power generation unit to charge 100 μF, 220 μF, and 330 μF capacitors under different external excitations, as depicted in Figure 5c (Figure 5c(i), (ii), (iii) show the voltages of different load capacitors under simulated wind energy excitation, wave energy excitation and wind-wave superposition excitationn, respectively). The results show that under the simulated wind-wave superposition excitation, a single power generation unit was able to charge the 100 μF, 220 μF, and 330 μF capacitors to 11.6 V, 4.57 V, and 3.34 V, respectively, in 100 s, presenting better charging capability. This also indicates that the higher output performance of power generation unit speeds up the charging rate of the capacitors under the superposition of two kinds of external excitation. In addition, Figure 5d(i), (ii), (iii) show output power and output current of a single power generation unit under simulated wind energy excitation, wave energy excitation and wind-wave superposition excitationn, respectively. The results show that the output current decreases and the output power first increases and then decreases as the load resistance increases, and the peak power is reached at around 10 mΩ for all three kinds of excitation. The peak power of a single power generation unit under three kinds of different excitation, namely, simulated sea wind excitation (4.5 m/s), simulated wave excitation (swing angle of 45° and frequency of 1.5 Hz), and simulated wind-wave superposition excitation, is 1.58 mW, 4.6 mW, and 5.9 mW, respectively. It is clear that the peak power under the simulated wind-wave superposition excitation exhibits a significant improvement of 273% and 28% when compared to that under simulated sea wind excitation or wave excitation, respectively. The above results prove that the WWS-TENG can realize the reverse motion process of two rotors of the generator driven by wind-wave energy, which improved the output power and accelerated charge transfer.

#### 3.3.4. The Output Performance of Two Power Generation Units in Parallel

**Figure 6:**
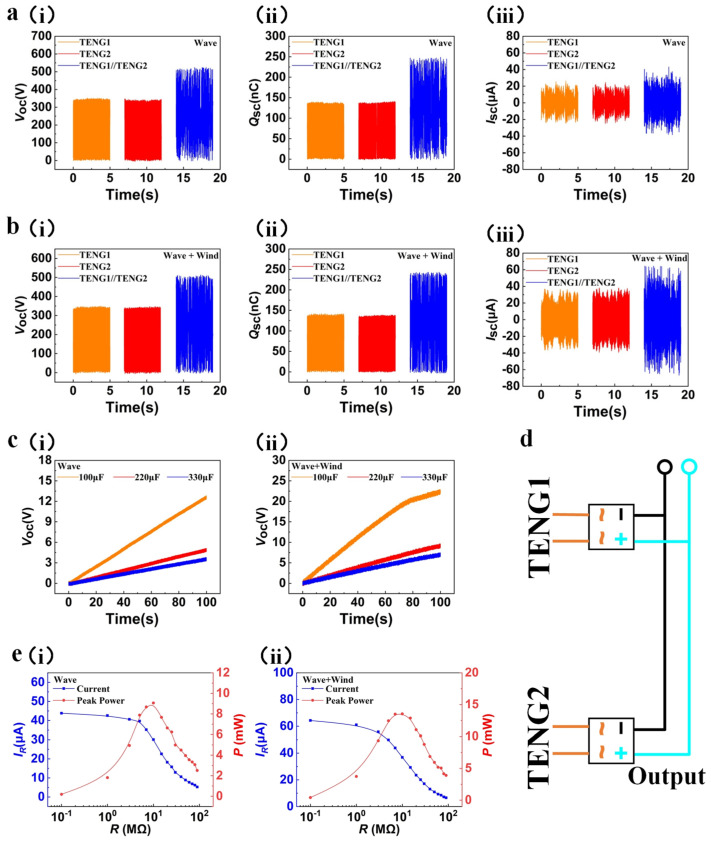
The output performance of two power generation units in parallel under simulated wind-wave superposition excitation: (**a**) the output performance of the WWS-TENG under simulated wave excitation; (**b**) the output performance of the WWS-TENG under simulated wind-wave superposition excitation; (**c**) voltage of different load capacitors under different external excitations; (**d**) the equivalent circuit of the two power generation units connected in parallel; (**e**) output power and output current of the WWS-TENG under different external excitations.

Since the wave-driven process can carry out power generation in the full time domain, we also experimentally compare wave power generation alone with wind-wave composite power generation under simulated conditions. The left and right side power generation units of the WWS-TENG were connected in parallel, and the simulated wind speed of the experiment was adjusted to 5.0 m/s, the swing angle of the swinging lever was adjusted to 45°, and the swing frequency was adjusted to 1.5 Hz. Figure 6a,b show the output performance of the WWS-TENG under simulated wave excitation and simulated wind-wave superposition excitation, respectively. Under simulated wave excitation, the open-circuit voltage (Figure 6a(i)) of the two power generation units in parallel is about 510 V, the transferred charge (Figure 6a(ii)) is about 240 nC, and the short-circuit current (Figure 6a(iii)) is about 43.1 μA, whereas under simulated wind-wave superposition excitation, the open-circuit voltage (Figure 6b(i)) and transferred charge (Figure 6b(ii)) are almost unchanged, while the short-circuit current (Figure 6b(iii)) rises to 64.5 μA.

We also study the output performance of two power generation units in parallel under the simulated wind-wave superposition excitation with different wind speeds (Figures S6–S8). In addition, we design the equivalent circuit of the two power generation units connected in parallel and rectified to charge capacitors of different capacities; then, we compare the charging capacity of the WWS-TENG under two kinds of external excitation for different capacitances, as depicted in Figure 6c,d (Figure 6c(i),(ii) show the voltages of different load capacitors under simulated wave energy excitation and wind-wave superposition excitationn, respectively). When charging a capacitor of the same capacity, the charging rate under simulated wind-wave superposition excitation is much faster than that under simulated wave excitation. For example, the WWS-TENG was able to charge a capacitor of 100 μF to 22.1 V in 100 s under simulated wind-wave superposition excitation, whereas it could only charge the capacitor to 12.5 V in the same 100 s under simulated wave excitation. Figure 6e(i),(ii) show output power and output current of WWS-TENG under simulated wave energy excitation and wind-wave superposition excitation, respectively. The maximum output power was achieved at a load resistance of around 10 MΩ for both kinds of external excitation. The peak power of the WWS-TENG is 9.06 mW under simulated wave excitation, and it is 13.5 mW under simulated wind-wave superposition excitation; the output of the WWS-TENG increased by 49% compared to single-wave power generation. 

#### 3.3.5. Application Demonstration

**Figure 7:**
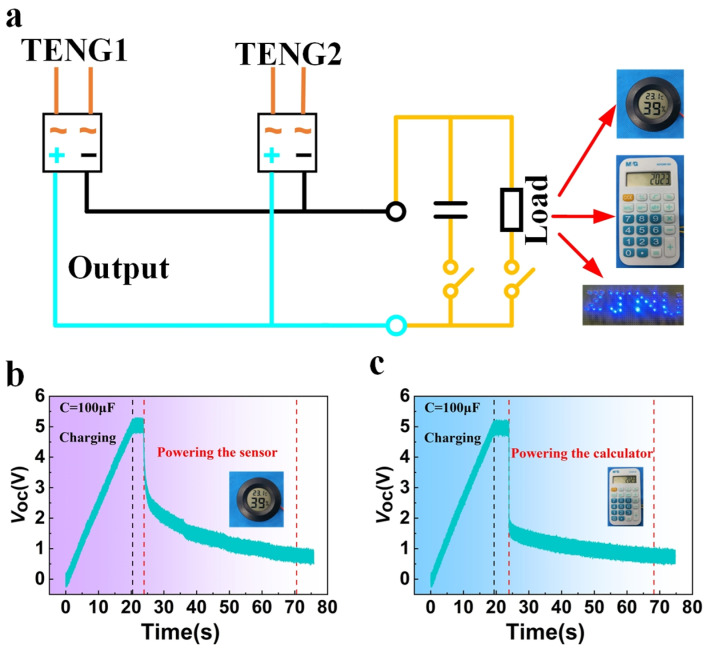
Application demonstrations of the WWS-TENG: (**a**) circuit diagram of the WWS-TENG for powering the sensor, calculator, and 36 LEDs; (**b**) the WWS-TENG powers the temperature/humidity sensor; (**c**) the WWS-TENG powers the calculator.

In order to verify the capability of the WWS-TENG in real-world applications, an experimental wind-wave simulation test bed was established, and simulated waves and wind were utilized as external excitation sources. The circuit diagram of the demonstration system is shown in Figure 7a, where a rectifier bridge is utilized to convert the WWS-TENG’s alternating current to direct current, and a 100 µF capacitor is used as a power storage unit to power low-power electronic devices. In this simulated environment, the WWS-TENG can directly light up 36 blue light-emitting diodes. As shown in Figure 7b, the position of the black dash line in the figure represents the time it takes for the WWS-TENG to charge the 100 µF capacitor, and the positions of the two red dash lines from left to right represent the positions where the capacitor starts to supply power to the sensor and stops supplying power after the WWS-TENG stops working, respectively. The WWS-TENG can charge the 100 µF capacitor to 5.2 V in about 20 s, and the sensor can continue working for about 45 s after the WWS-TENG stops working. We also considered stability and durability issues for powering low-power sensors. By using the thinner FEP blades and the acrylic shells for sealing, the surface wear of the copper electrode is lower, and the degree of moisture affecting the power generation unit has significantly decreased. In durability and stability tests lasting about a week, the WWS-TENG maintained a relatively stable charging rate for powering the calculator, as shown in Figure 7c, showing its good stability and durability in powering low-power sensors. This also demonstrates the capability of the WWS-TENG in supplying power to low-power sensors and highlights its potential application in the Internet of Things (IoT).

## 4. Conclusions

In a summary, we designed and fabricated a wind-wave synergistic triboelectric nanogenerator (WWS-TENG) that collects wave energy and sea wind energy in marine environments. The WWS-TENG is designed according to the characteristics of the marine environment. Thanks to the transmission mechanism, wind and wave excitation are able to drive the two rotors of the generator to rotate in opposite directions, respectively, which increases the relative rotational speed of the rotors, accelerates the charge transfer rate, improves the output performance, and realizes the simultaneous collection of sea wind energy and wave energy. The output performance of a single power generation unit of the WWS-TENG was tested in a simulated marine environment, and the effects of wind speed, swing angle, and frequency on its energy harvesting were investigated. When the wind speed of the experiment was adjusted to 5.0 m/s, the swing angle of the swinging lever was adjusted to 45°, and the swing frequency was adjusted to 1.5 Hz. The open-circuit voltage of the WWS-TENG can reach about 510 V, the transferred charge is about 240 nC, the maximum short-circuit current is about 64.5 μA, and the peak power can reach 13.5 mW under simulated wind-wave superposition excitation. In addition, the WWS-TENG could directly power multiple LED lights, charge commercial capacitors, and successfully power a calculator and temperature/humidity sensor, demonstrating its good power generation capability. This work has achieved the coordinated generation of wind and waves using TENG, which will further promote the development of offshore monitoring and blue energy utilization.

## Data Availability

Data are contained within the article and Supplementary Materials.

## Supplementary Materials

The following supporting information can be downloaded at https://www.mdpi.com/article/10.3390/mi15030314/s1: Figure S1: Force analysis of the energy storage stage; Figure S2: Force analysis of the energy release stage; Figure S3: Comparison of output voltage curves under intermittent rotation and continuous rotation; Figure S4: Comparison of transferred charge curves under intermittent rotation and continuous rotation; Figure S5: Comparison of output current curves under intermittent rotation and continuous rotation; Figure S6: The open-circuit voltage of the WWS-TENG under the simulated wind-wave superposition excitation with different wind speeds; Figure S7: The transferred charge of the WWS-TENG under the simulated wind-wave superposition excitation with different wind speeds; Figure S8: The short-circuit current of the WWS-TENG under the simulated wind-wave superposition excitation with different wind speeds. Supplemental Notes: Note S1: The moment balance equation of the energy storage stage; Note S2: The moment balance equation of the energy release stage.
