# Supplementary material for: Wind-Wave Synergistic Triboelectric Nanogenerator: Performance Evaluation Test and Potential Applications in Offshore Areas
Source: Micromachines (Basel). 2024 Feb 24;15(3):314. doi: 10.3390/mi15030314 (PMC10972333; doi:10.3390/mi15030314)
Supplement: Supplementary file 1 [file micromachines-15-00314-s001.zip › micromachines-2862048-supplementary.pdf]

Supporting Information

Wind-Wave Synergistic Triboelectric Nanogenerator：Performance Evaluation Test and Potential Applications in Offshore Areas

Zhen Pan [1], Weijian Wu [1], Jiangtao Zhou [1], YiLi Hu [1], Jianping Li [1], Yingting Wang [1,*], Jijie Ma [1, 2,*] and Jianming Wen [1, 2,*]

1 The Institute of Precision Machinery and Smart Structure, College of Engineering, Zhejiang Normal University, Yinbin Street 688, Jinhua, 321004, China
2 Key Laboratory of Intelligent Operation and Maintenance Technology & Equipment for Urban Rail Transit of Zhejiang Province, Zhejiang Normal University, Yinbin Street 688, Jinhua, 321004, China
* Correspondence: wangyingting@zjnu.edu.cn, mjj@zjnu.cn, wjming@zjnu.cn

Content

Supplemental Figures

Figure S1. Force analysis of the energy storage stage
Figure S2. Force analysis of the energy release stage
Figure S3. Comparison of output voltage curves under intermittent rotation and continuous rotation
Figure S4. Comparison of transferred charge curves under intermittent rotation and continuous rotation
Figure S5. Comparison of output current curves under intermittent rotation and continuous rotation
Figure S6. The open-circuit voltage of WWS-TENG under the simulated wind-wave superposition excitation with different wind speeds
Figure S7. The transferred charge of WWS-TENG under the simulated wind-wave superposition excitation with different wind speeds
Figure S8. The short-circuit current of WWS-TENG under the simulated wind-wave superposition excitation with different wind speeds

Supplemental Notes

Note S1. The moment balance equation of the energy storage stage
Note S2. The moment balance equation of the energy release stage

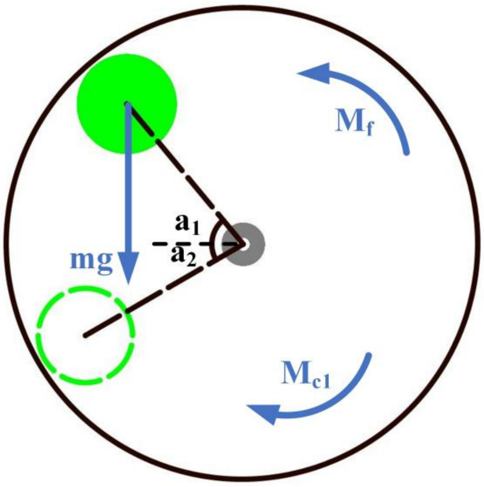

Figure S1. Force analysis of the energy storage stage

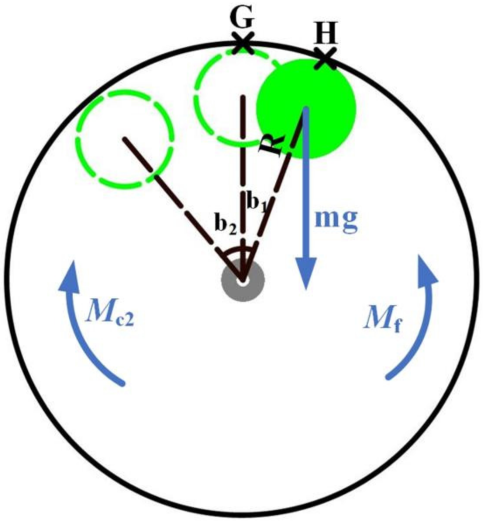

Figure S2. Force analysis of the energy release stage

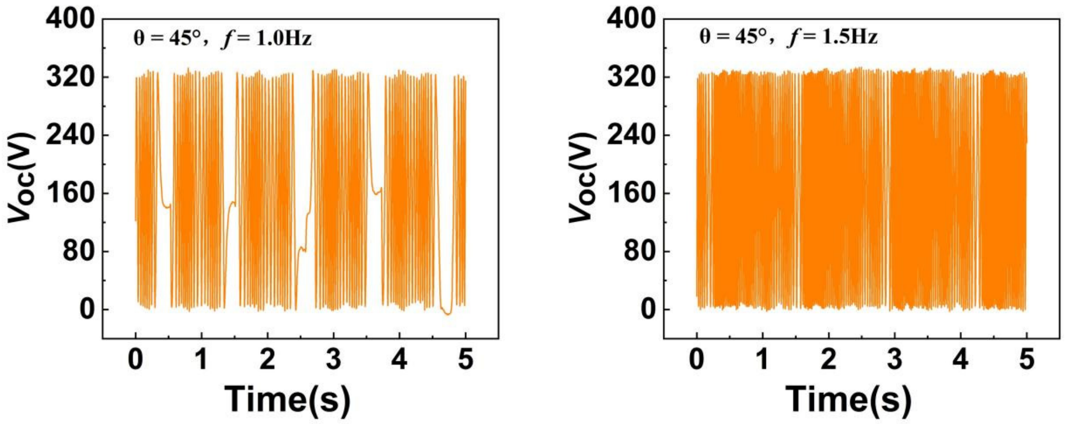

Figure S3. Comparison of output voltage curves under intermittent rotation and

continuous rotation

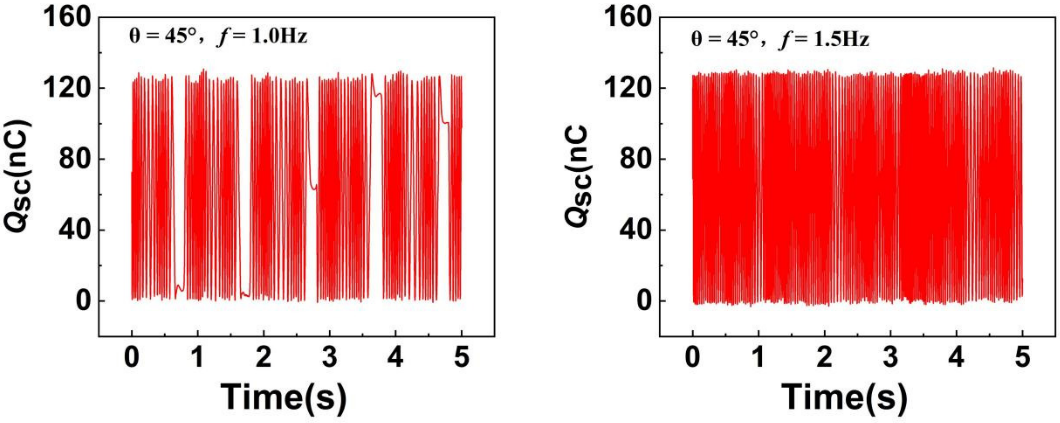

Figure S4. Comparison of transferred charge curves under intermittent rotation and continuous rotation

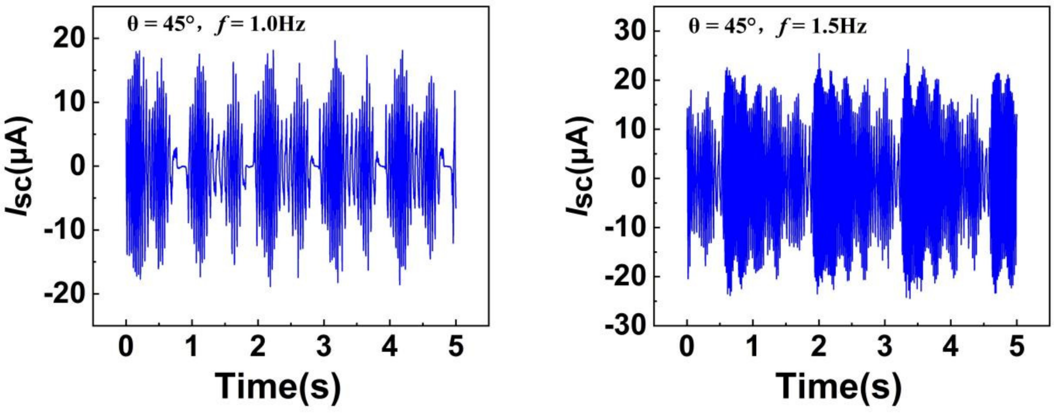

Figure S5. Comparison of output current curves under intermittent rotation and continuous rotation

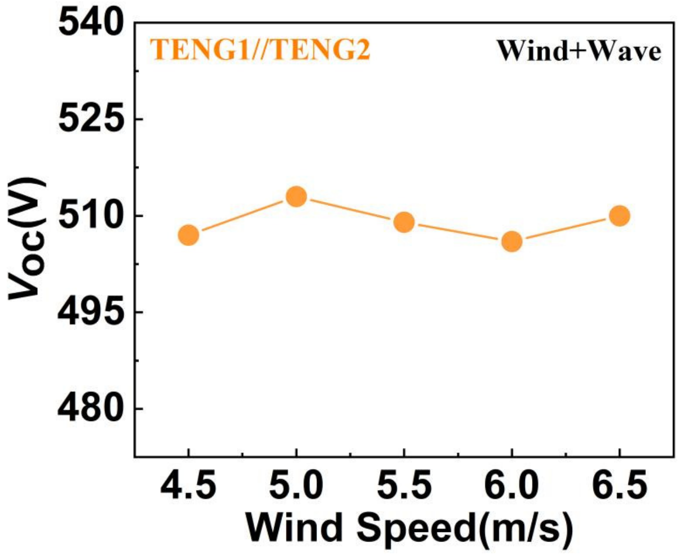

Figure S6. The open-circuit voltage of WWS-TENG under the simulated wind-wave superposition excitation with different wind speeds

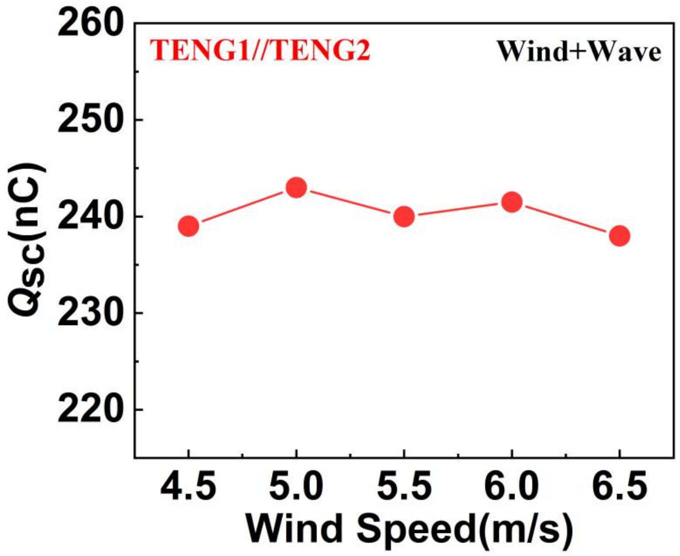

Figure S7. The transferred charge of WWS-TENG under the simulated wind-wave superposition excitation with different wind speeds

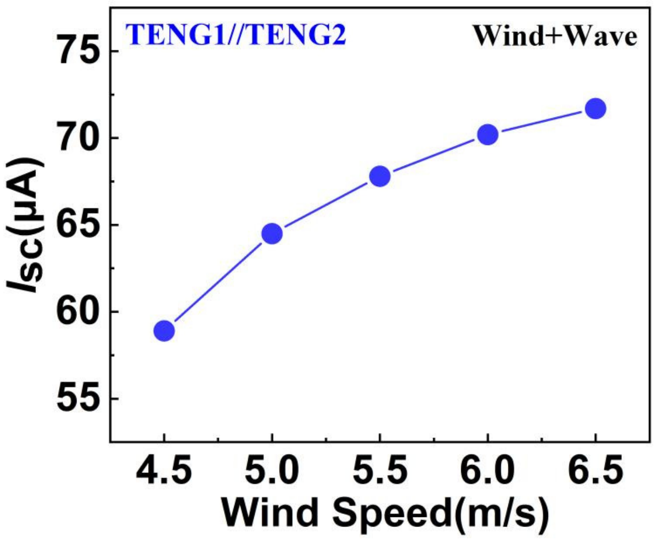

Figure S8. The short-circuit current of WWS-TENG under the simulated wind-wave superposition excitation with different wind speeds

Note S1. The moment balance equation of the energy storage stage

Based on force analysis in **Figure** S1, the moment balance equation of the center shaft on which the acrylic disc with FEP and mass block is fixed is as follows:

$$J\frac{d^2(a_1+a_2)}{dt_1{}^2}=M_{c1}-\mathrm{Rmgcos}a_1-M_f \tag{S1}$$

Where J is rotational inertia of the center shaft on which the acrylic disc with FEP and mass block, R is the distance from the center of rotation to the center of the mass block, the angular displacement of acrylic disc with FEP and mass block is divided into angle a_1 and a_2 by a horizontal line, g is the gravitational acceleration, m is the weight of the mass block, M_f is the moment of friction force on the the acrylic disc with FEP and mass block, and M_{c1} is the moment acting on this center shaft.

Note S2. The moment balance equation of the energy release stage

Based on force analysis in **Figure** S2, the moment balance equation of the center shaft on which the acrylic disc with FEP and mass block is fixed is as follows:

$$J\frac{d^2(b_1+b_2)}{dt_2{}^2}=M_{c2}+\mathrm{Rmgsin}b_1-M_f \tag{S2}$$

Where J , R, g, m and M_f are depicted in **Note S1**, the angular displacement of acrylic disc with FEP and mass block is divided into angle b_1 and b_2 by a vertical line, M_{c2} is the moment acting on this center shaft.
